# First Observation of Hemoglobin San Diego, a High Oxygen Affinity Hemoglobin Variant, in Turkey

**DOI:** 10.4274/tjh.2017.0213

**Published:** 2017-12-01

**Authors:** Ebru Yılmaz Keskin, Ali Fettah, Ana Catarina Oliveira, Şule Toprak, Andreia Lopes, Celeste Bento

**Affiliations:** 1 Süleyman Demirel University Faculty of Medicine, Department of Pediatric Hematology and Oncology, Isparta, Turkey; 2 Coimbra University, Centro Hospital, Clinic of Hematology, Coimbra, Portugal; 3 CIAS, Coimbra University, Department of Life Sciences, Coimbra, Portugal

**Keywords:** Abnormal hemoglobins, Hemoglobin San Diego, Hemoglobin variant

## To The Editor,

Congenital erythrocytosis (CE) or congenital polycythemia represents a rare clinical entity. High oxygen affinity hemoglobin (Hb) variants are a very rare cause of secondary CE. In 1966, Charache et al. [1] published the first case of a Hb variant associated with erythrocytosis, Hb Chesapeake. Since then, more than 220 variants with high oxygen affinity have been discovered and the autosomal dominant inheritance has been confirmed [2].

Many Hb variants have been reported so far from Turkey [3,4,5]. We report herein the first observation of Hb San Diego, a high oxygen affinity Hb variant, from Turkey in a case of CE.

**Figure 1:**
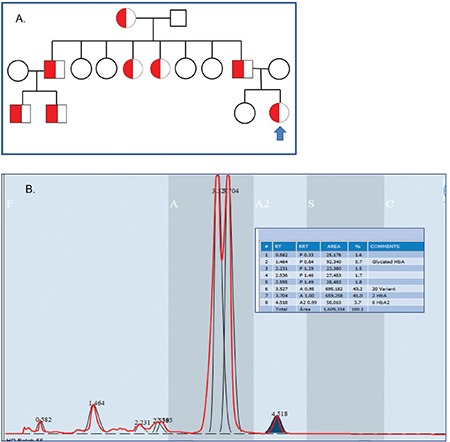
A) Pedigree of the family with erythrocytosis and hemoglobin (Hb) San Diego, illustrating dominant mode of inheritance of erythrocytosis. The propositus is indicated with an arrow; B) high-performance liquid chromatography (premier Hb9210 resolution) showing the presence of Hb San Diego.

Case: A 15-year-old female patient residing in Kastamonu, Turkey, and examined due to the complaints of occasional headache, fatigue, dizziness, nausea, and chest pain was found to have an elevated Hb level. Erythrocytosis was also present in other family members, including her father and paternal grandmother (Figure 1). Both the father and grandmother had a history of several phlebotomies.

**Figure 2:**
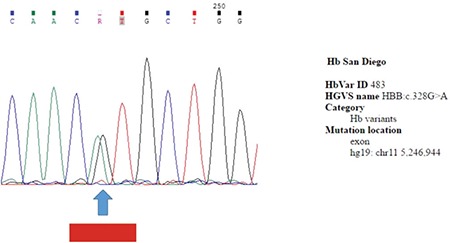
Identification of hemoglobin San Diego in β-globin gene by Sanger sequencing analysis in the index case. HbVar: Hemoglobin variant.

Laboratory data are presented in Table 1. Serum biochemistry, abdominal ultrasonography, and echocardiographic examinations were all unremarkable. In addition to her family history consistent with a disorder transmitted autosomal dominantly, the finding of reduced P50 suggested the presence of a high oxygen affinity Hb. Hb electrophoresis performed with the high-performance liquid chromatography (HPLC) method with the device ZIVAK using the Hb Variant Whole Blood HPLC Analysis Kit yielded no abnormal Hb variant. The examination was repeated with Trinity Biotech’s Premier Hb9210 resolution method and displayed the presence of a Hb variant in both the patient and her father (Figure 1). Sanger sequencing analysis confirmed the associated mutation in the β-globin gene [Hb San Diego; β109(G11)Val→Met] (Figure 2).

Erythrocytosis may be the clinical manifestation of the presence of a high oxygen affinity Hb. Hb San Diego was first reported in 1974 in a Filipino family [6]. Thereafter, it has been described in subjects of different origins [7,8,9,10,11,12]. Our case represents the first one of Hb San Diego in Turkey. Although Hb San Diego was described as electrophoretically silent [6], it could be clearly identified using the new Trinity Biotech Premier Hb9210 resolution technology.

In their study evaluating 70 patients with CE, Bento et al. [11] sequenced all the genes described as associated with CE and erythrocytosis molecular etiology was identified in only 25 (36%) subjects, a high oxygen affinity Hb being the cause in 14 (56%) of these 25 subjects. Determination of the P50 value, calculated easily from fresh venous blood gas samples, is a practical and useful test, and a decreased value may direct clinicians to order examinations regarding a Hb variant [12]. Some high oxygen affinity Hbs are electrophoretically silent but their identification can be rapidly done by direct sequencing of the globin genes (HBB and HBA).

Management of CE caused by a high oxygen affinity Hb should be personalized, and it should primarily depend on smoking cessation and physical activity. Phlebotomy and platelet aggregation inhibitors’ prescription should be evaluated carefully, and blood donation is not advised [2].

## Figures and Tables

**Table 1:**
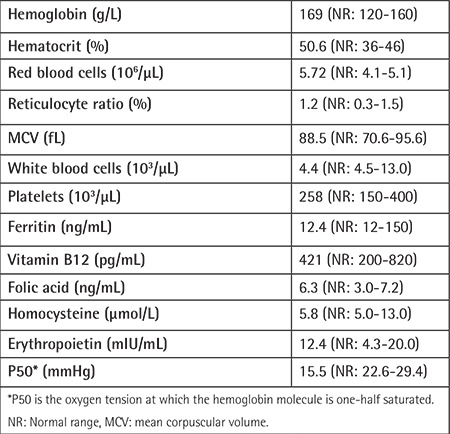
Laboratory findings of the patient at the time of admission.
